# Lipid droplet dynamics and insulin sensitivity upon a 5-day high-fat diet in Caucasians and South Asians

**DOI:** 10.1038/srep42393

**Published:** 2017-02-14

**Authors:** Anne Gemmink, Leontine E. H. Bakker, Bruno Guigas, Esther Kornips, Gert Schaart, A. Edo Meinders, Ingrid M. Jazet, Matthijs K. C. Hesselink

**Affiliations:** 1 Department of Human Biology and Human Movement Sciences, NUTRIM School of Nutrition and Translational Research in Metabolism, Maastricht University Medical Centre+, 6200 MD, Maastricht, The Netherlands; 2 Department of Internal Medicine, section Endocrinology, Leiden University Medical Center, The Netherlands; 3 Department of Molecular Cell Biology, Leiden University Medical Center, The Netherlands; 4 Department of Parasitology, Leiden University Medical Center, The Netherlands

## Abstract

A 5-day High-Fat High-Calorie diet (HFHC-diet) reduces insulin-stimulated glucose disposal (Rd) in South Asian, but not Caucasian healthy lean males. We aimed to investigate if differences in myocellular lipid handling are underlying this differential response. A two-step hyperinsulinemic-euglycemic clamp and muscle biopsies were performed in 12 healthy lean Caucasian and South Asian males (BMI < 25 kg/m^2^, 19–25 years) before and after a 5-day HFHC-diet (regular diet + 375 mL cream/day; 1275 kcal/day; 94% fat). Triglyceride extractions and Western Blots for lipid droplet and mitochondrial proteins were performed. Intramyocellular lipid content and HFHC-diet response were similar between ethnicities (group effect: P = 0.094; diet effect: +~30%, P = 0.044). PLIN5 protein content increased upon the HFHC-diet (P = 0.031) and tended to be higher in South Asians (0.87 ± 0.42 AU vs. 1.35 ± 0.58 AU, P = 0.07). 4-HNE tended to increase in South Asians upon the HFHC-diet (interaction effect: P = 0.057). In Caucasians ΔPLIN5 content correlated with ΔR_d_ (Caucasians: r = 0.756, P = 0.011; South Asians: r = −0.085, P = 0.816), while in South Asians Δ4-HNE associated with ΔPLIN5 content (Caucasians: r = 0.312, P = 0.380; South Asians: r = 0.771, P = 0.003). These data indicate that in Caucasians, PLIN5 may be protective against HFHC-diet induced insulin resistance, which for reasons not yet understood is not observed in South Asians, who possess increased lipid peroxidation levels.

The prevalence of type 2 diabetes is rising worldwide, more particularly in the South Asian population^1^. Compared to the Caucasian population South Asians carry a number of additional risk factors for developing type 2 diabetes. South Asians have a higher percentage body fat at a similar BMI^2^ and store more of their fat in visceral adipose tissue compartments compared to Caucasians^3^,^4^.

Interestingly, at the same BMI South Asians also store more fat as intramyocellular lipids (IMCL), compared to Caucasians^5^,^6^. In the Caucasian population a negative association between IMCL content and insulin sensitivity has been reported^7^,^8^. This has led to the suggestion that lipids stored in skeletal muscle impede insulin sensitivity. However, observations in trained athletes, who also store muscle fat quite abundantly whilst being rather insulin sensitive, indicate that it is unlikely that this association is causal^9^. Fascinatingly, also in the South Asian population the relationship between IMCL content and insulin resistance is not observed^5^,^10^.

These observations in two phenotypically different populations indicate that the amount of IMCL is not necessarily a determinant of insulin sensitivity. Rather, the capacity of the IMCL pool to undergo continuous release and storage of fat in order to meet changes in demand and supply, a process referred to as lipid droplet (LD) dynamics, might be of more importance for insulin sensitivity^11^ than IMCL content *per se*. Thus in a state of reduced LD dynamics, IMCL may impede insulin sensitivity more profoundly. Although mitochondrial content^3^,^12^ and function^6^ in South Asians are similar or even higher compared to Caucasians, fat oxidative capacity during submaximal exercise is lower in South Asians than in Caucasians^3^. This observation may indicate that release of fatty acids to fuel oxidation in South Asians may be compromised and that LDs in skeletal muscle of South Asians are less dynamic than in Caucasians.

Recognized players in skeletal muscle LD dynamics are proteins from the perilipin family (PLIN1-5), the major adipose triacylglycerol lipase ATGL^13^ and its co-activator CGI-58^14^. While ATGL and CGI-58 jointly stimulate LD lipolysis, PLIN5 regulates LD lipolysis by interacting with ATGL and CGI-58^15^ to facilitate fat oxidation. Insulin sensitive endurance trained athletes, recognized for the highly dynamic nature of their LD pool, possess higher expression of PLIN2, PLIN5 and ATGL compared to lean untrained individuals^16^. In addition training induces ATGL, PLIN3 and PLIN5 protein expression in obese subjects^17^. Upon consumption of a high fat diet, IMCL content increases in rodents while ATGL drops^18^ and expression of PLIN5^18^,^19^ and CGI-58 increases^18^, also indicative of alterations in LD dynamics. Thus, both insulin sensitizing and desensitizing interventions affect proteins involved in LD dynamics in skeletal muscle.

We recently published that upon a 5-day high-fat, high-calorie diet (HFHC-diet) South Asians develop peripheral insulin resistance, while Caucasians remain insulin sensitive^12^. Increases in IMCL content with reduced insulin sensitivity have been reported upon short term high-fat diets up to 3 days^20^,^21^. When high-fat diets are maintained for a longer period, the effects on IMCL and insulin sensitivity are less prominent, if not absent^22^,^23^,^24^,^25^. Here we used the 5-day HFHC-diet approach to examine the role of IMCL and LD dynamics in the differential response in insulin sensitivity of Caucasians and South Asians. We hypothesized that Caucasians are better capable to maintain insulin sensitivity upon a lipid overload due to differences in LD dynamics than South Asians. To test this hypothesis we analyzed muscle biopsies of South Asians and Caucasians on IMCL content and expression of proteins involved in LD dynamics before and after the 5-day HFHC-diet.

## Results

### Subject characteristics

The subject characteristics were previously reported elsewhere^12^. In short, at baseline South Asians had a higher fat mass, HbA_1c_ and higher insulin levels during the clamp than Caucasians. Metabolic flexibility and peripheral insulin sensitivity (Rd) was similar for both ethnicities at baseline. The 5-day HFHC-diet induced a very modest weight gain in both groups (Caucasians: from 75.1 ± 1.8 to 75.6 ± 1.8 kg, not significant; South Asians: from 63.2 ± 2.3 to 63.7 ± 2.3 kg, P < 0.05) and insulin resistance in South Asians, but not in Caucasian subjects (Caucasians: from 41.7 ± 2.9 to 41.0 ± 2.8 μmol/kg_LBM_/min, not significant; South Asians: from 48.7 ± 2.9 to 39.0 ± 2.1 μmol/kg_LBM_/min, P < 0.005)^12^.

### PLIN5 protein content associates with peripheral insulin sensitivity in Caucasians and not in South Asians at baseline

**Figure 1:**
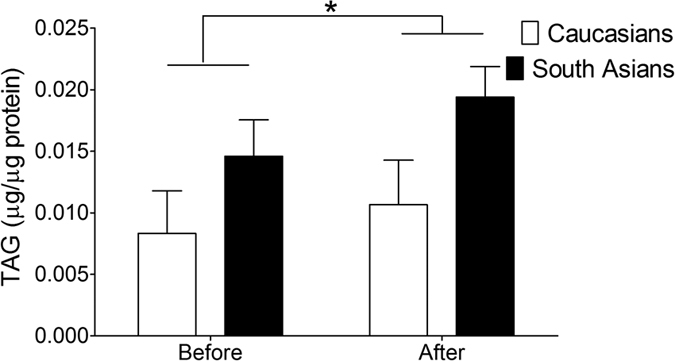
Intramyocellular lipid content (triacylglycerol, TAG) in Caucasian and South Asian subjects before and after a 5-day HFHC-diet. Data are presented as mean ± SEM and were statistically analyzed with a Repeated Measures ANOVA; *P < 0.05 for diet effect.

**Figure 2:**
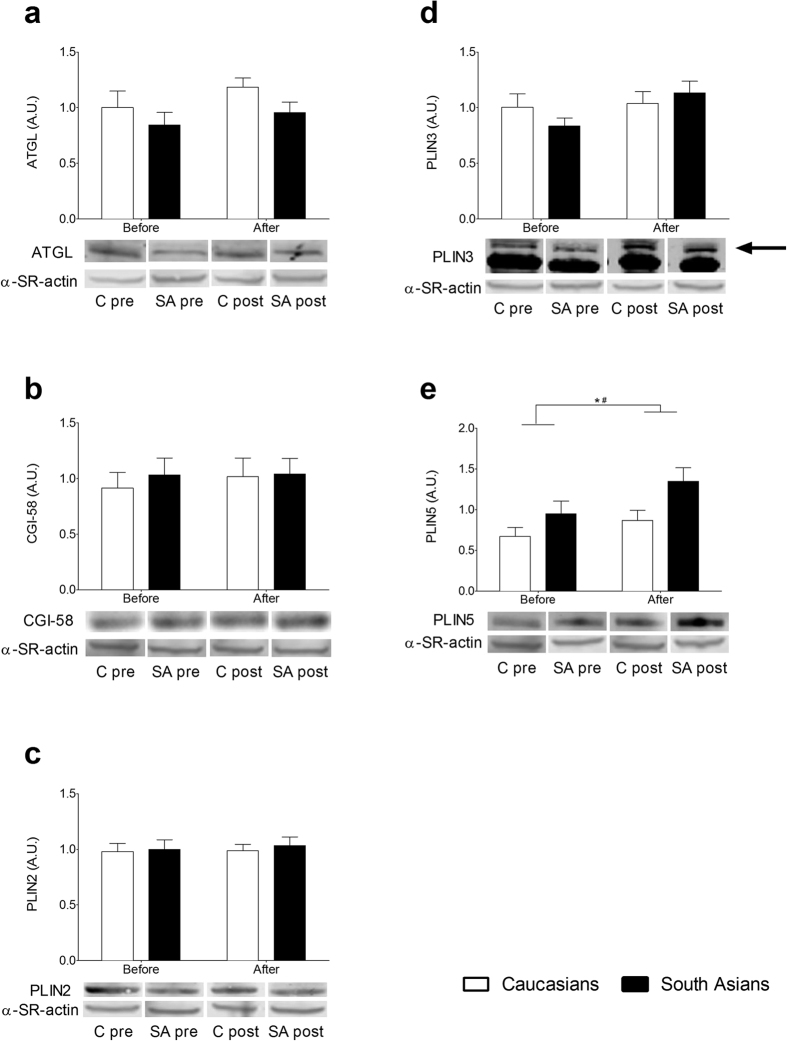
Protein content of important players involved in lipid droplet dynamics in Caucasian and South Asian subjects before and after a 5-day HFHC-diet. (**a**) ATGL, (**b**) CGI-58, (**c**) PLIN2, (**d**) PLIN3 and (**e**) PLIN5 protein content. Data are presented as mean ± SEM and were statistically analyzed with a Repeated Measures ANOVA; *P < 0.05 for diet effect, ^#^P < 0.05 for group effect.

We examined baseline IMCL content and protein expression of ATGL, CGI-58, PLIN2, PLIN3 and PLIN5 to assess putative differences between Caucasians and South Asians. As shown in Fig. 1, baseline IMCL content was not significantly different between ethnicities (0.008 ± 0.003 *vs.* 0.015 ± 0.003 μg/μg protein for Caucasians and South Asians, respectively). Furthermore, protein contents of ATGL, CGI-58, PLIN2, PLIN3 and PLIN5 were comparable in Caucasians and South Asians (ATGL: 1.00 ± 0.15 vs. 0.84 ± 0.11 AU, CGI-58: 0.91 ± 0.14 vs. 1.03 ± 0.15 AU, PLIN2: 0.98 ± 0.07 vs.1.00 ± 0.09 AU, PLIN3: 1.00 ± 0.12 vs. 0.84 ± 0.07 AU, PLIN5: 0.67 ± 0.11 vs. 0.95 ± 0.16 AU for Caucasians vs. South Asians; Fig. 2).

Of these proteins, PLIN5 has been associated with changes in (markers of) lipid-related insulin resistance^26^,^27^,^28^. Given the fairly wide range in insulin sensitivity within the groups, we anticipated that correlation analysis of PLIN5 with Rd could yield relevant information, even though the primary aim of the study was the comparison between Caucasians and South Asians. Thus, we assessed whether baseline IMCL content and protein levels of ATGL, CGI-58, PLIN2, PLIN3 and PLIN5 correlated with Rd. IMCL, ATGL, PLIN2 and PLIN3 content did not correlate with Rd, neither in Caucasians nor in South Asians (data not shown). In South Asians, however, CGI-58 correlated positively with Rd (r = 0.749, P = 0.013) whereas no such correlation was observed in Caucasians (r = −0.186, P = 0.585). Interestingly, PLIN5 also correlated positively with Rd in Caucasian subjects, but not in South Asian subjects (Table 1). In support of this association we observed positive correlations between PLIN5 and other surrogate markers of insulin sensitivity (insulin stimulated RQ, metabolic flexibility, insulin-stimulated glucose oxidation and insulin stimulated NOGD, Table 1 and Supplementary Fig. S1).

### Response of IMCL and PLIN5 to a 5-day HFHC-diet

We previously reported that the 5-day HFHC-diet induced insulin resistance in South Asians, whereas Caucasians were capable of maintaining peripheral insulin sensitivity^12^. This differential response of insulin sensitivity to a HFHC-diet might be due to a different response of IMCL and/or lipid droplet coating proteins to the HFHC-diet.

Although we did not observe a significant interaction effect (P = 0.456) for IMCL content, there was a significant diet effect (P = 0.044) with a tendency for a group effect (P = 0.094). In both ethnicities IMCL content increased ~30% upon the HFHC-diet (Fig. 1). While protein contents of ATGL, CGI-58 and PLIN2 were unaffected by the HFHC-diet and did not differ between groups after the diet (Fig. 2), a tendency for an increase in PLIN3 protein content was observed (diet effect, P = 0.074). For PLIN5, however, we observed a significant group effect (P = 0.027), which tended to be higher after the HFHC-diet in the South Asians (0.87 ± 0.13 AU and 1.35 ± 0.17 AU for Caucasians and South Asians respectively, P = 0.07). We observed a diet effect (P = 0.031) for PLIN5 as well, with a comparable diet-induced increase in PLIN5 for both groups (23% for Caucasians and 49% for South Asians, P = 0.430).

### PLIN5 correlates with insulin sensitivity in Caucasians after the 5-day HFHC-diet

As we observed a positive correlation between PLIN5 content and markers of insulin sensitivity at baseline in the Caucasian subjects, we also explored if this correlation was maintained after consumption of the HFHC-diet. In line with the observation prior to the HFHC-diet, IMCL content was not associated with Rd after the diet, irrespective of ethnicity (r = −0.247, P = 0.522 for Caucasians and r = −0.408, P = 0.213 for South Asians). In addition, the HFHC-diet induced change in IMCL content did not correlate with the change in Rd (r = −0.064, P = 0.918 for Caucasians and r = −0.517, P = 0.190 for South Asians).

**Figure 3:**
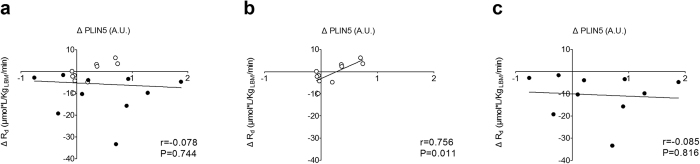
Correlations between the HFHC-diet induced changes in PLIN5 protein content and glucose disposal rate. (**a**) All participants, (**b**) Caucasian participants and (**c**) South Asian participants. Pearson correlation coefficients were used to describe the linear associations; Caucasians: ○, South Asians: ●.

Similar to our observations before the HFHC-diet, PLIN5 protein content correlated positively with Rd and insulin-stimulated NOGD in Caucasians (Table 1 and Supplementary Fig. S2), but not in South Asians after the diet. Interestingly, when we correlated the change in PLIN5 content with the changes in Rd we did not observe a correlation for all subjects together (South Asians and Caucasians) (Fig. 3a, r = −0.078, P = 0.744). However, in the Caucasian participants we did observe a positive correlation between the change in PLIN5 content and the change in Rd (Fig. 3b, r = 0.756, P = 0.011), whereas no correlation was found in South Asians (Fig. 3c, r = −0.085; P = 0.816).

### Mitochondrial content and lipid peroxidation

**Figure 4:**
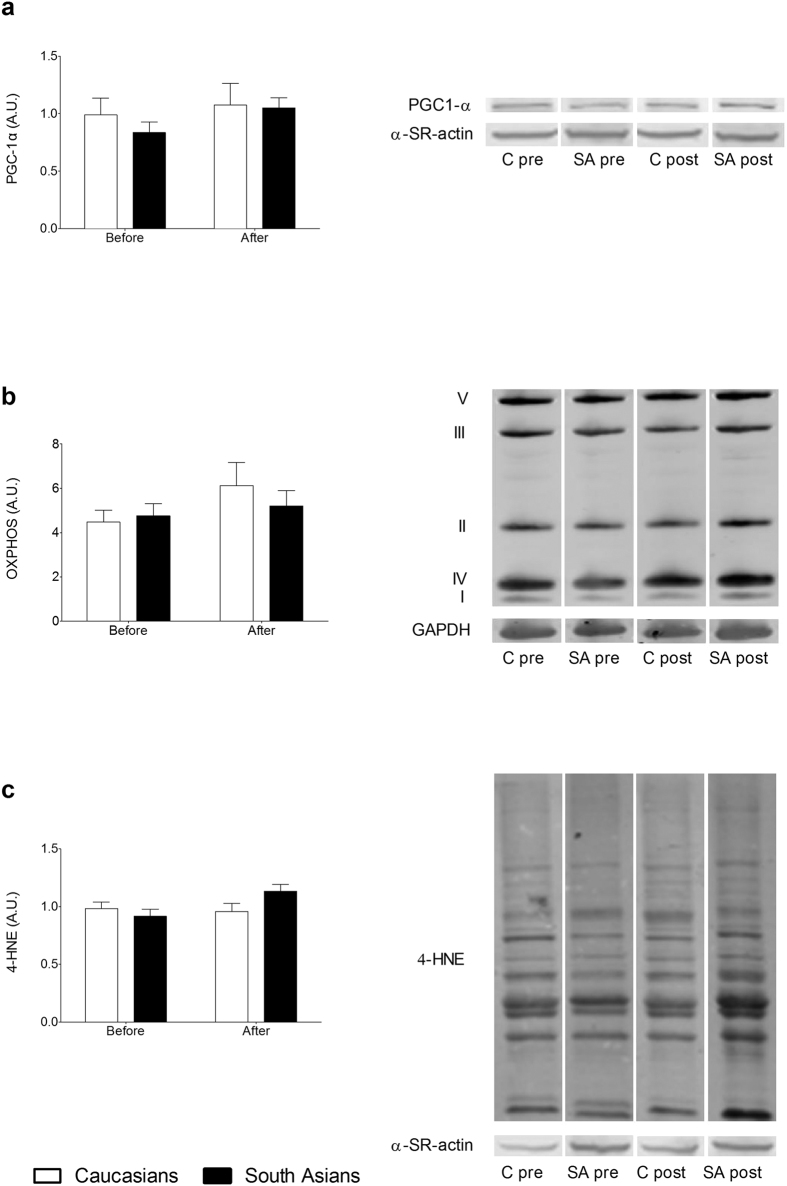
Mitochondrial content and lipotoxicity. Mitochondrial biogenesis (**a**), mitochondrial content measured as the total content of all 5 complexes (**b**) and levels of lipid peroxidation determined by 4-HNE (**c**) in Caucasian and South Asian subjects before and after a 5-day HFHC-diet. Data are presented as mean ± SEM and were statistically analyzed with a Repeated Measures ANOVA.

**Figure 5:**
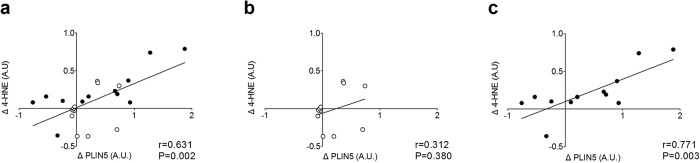
Correlations between the HFHC-diet induced changes in PLIN5 protein content and lipid peroxidation levels. (**a**) All participants, (**b**) Caucasian participants and (**c**) South Asian participants. Pearson correlation coefficients were used to describe the linear associations; Caucasians: ○, South Asians: ●.

In previous studies high PLIN5 protein levels have been associated with higher levels of insulin sensitivity^16^,^26^ and was reportedly to be protective against development of insulin resistance^27^,^28^. It is hence remarkable that South Asians developed insulin resistance upon a HFHC-diet, despite having higher levels of PLIN5 protein than Caucasians. PLIN5 is suggested to regulate LD dynamics for oxidative lipolysis. Thus, if fatty acids are released from the LD under conditions of compromised mitochondrial biogenesis or content, lipotoxicity and subsequently insulin resistance may develop. To explore this hypothesis, we measured marker proteins of mitochondrial biogenesis (PGC-1α) and content (various ETC complex subunits) along with a marker of lipid peroxidation, 4-hydroxynonenal (4-HNE). PGC-1α protein content was similar for South Asians and Caucasians before and after the HFHC-diet and was unaffected by the HFHC-diet (Fig. 4a). Mitochondrial content measured as total content of 5 different ETC complex subunits was similar between ethnicities at baseline and tended to increase mainly in the Caucasian subjects (diet effect P = 0.075) (Fig. 4b). Both the pattern of lipid peroxidation (Fig. 4c) as well as total 4-HNE content (Fig. 4d) was similar between ethnicities at baseline, whereas a tendency for an interaction effect (P = 0.057) was observed for 4-HNE content. Indeed, skeletal muscle 4-HNE content was unaltered in Caucasians upon the HFHC-diet whereas it tended to increase in South Asians. In addition, this increase in 4-HNE correlated positively with the increase in PLIN5 protein content in South Asians (r = 0.771, P = 0.003), but not in the Caucasians (r = 0.312, P = 0.380) (Fig. 5).

## Discussion

In the present study we aimed to examine whether the differential response on peripheral insulin sensitivity to a 5-day HFHC-diet between subjects from Caucasian and South Asian origin could be explained by differences in players involved in myocellular lipid handling. Although IMCL content increased similarly in both groups upon the HFHC-diet, subjects from South Asian origin possess more severe insulin resistance than Caucasians. None of the recognized players in LD dynamics (ATGL, CGI-58, PLIN2 and PLIN3) were significantly different between ethnicities at baseline, nor did they respond differentially to the HFHC-diet. So the more profound insulin resistance observed in South Asians could not be attributed to differences in LD dynamics. Surprisingly, the LD coat protein PLIN5, which is most commonly associated with improvements in insulin sensitivity or protection against lipid-induced insulin resistance, was found to be increased upon the HFHC-diet in both groups with a more robust effect in (the more insulin resistant) South Asians. The change in PLIN5 protein content correlated with the change in Rd in Caucasians. This matches the notion that PLIN5 might be considered as a LD coat protein associated with benign myocellular lipid storage. For reasons yet unknown, this correlation was absent in South Asians.

Whereas South Asians and Caucasians both increased IMCL content upon a 5-day HFHC-diet, only the South Asians became insulin resistant^12^. This may indicate that handling of excess lipids in South Asians may differ from Caucasians. Myocellular lipids are stored in LDs which are currently recognized as dynamic organelles that can readily switch between release and storage of fatty acids upon changes in substrate availability and demand. Release of fatty acids from myocellular LDs serves a dual role: fueling mitochondrial oxidation and/or acting as ligands for PPARα- and PGC1α-mediated changes in oxidative gene expression and mitochondrial biogenesis^16^,^29^,^30^. Liberation of fatty acids from the LD depends on the activation of ATGL^13^, partly via interaction with its co-factor CGI-58^14^ and PLIN5^31^. In muscle cells, PLIN5 binds to GCI-58 to suppress basal lipolysis^32^,^33^. Activation of lipolysis involves dissociation of PLIN5 and CGI-58, making CGI-58 available for interaction with ATGL to stimulate its lipolytic activity. Endogenously available long-chain Acyl-CoA esters can stimulate the association of PLIN5 and CGI-58 and thus prevents binding of CGI-58 with ATGL, hence preventing lipolysis^34^. Thus, the lipolytic rate of PLIN5-coated LDs can be tuned to substrate availability and demand, and levels of LD-derived insulin desensitizing lipids can be well-controlled, providing a mechanistic explanation on how PLIN5 can modulate lipid-induced insulin resistance. In accordance with this role of PLIN5, we previously observed that upon prolonged fasting PLIN5 was protective against fasting-induced insulin resistance^28^ whereas others have shown that PLIN5 indeed may protect against myocellular lipotoxicity^35^. In contrast, studies in PLIN5 null-mice revealed that PLIN5 is dispensable for normal substrate selection^36^. These studies, along with the current observation in Caucasians that those who increase PLIN5 content the most dropped the least in insulin sensitivity upon the HFHC-diet, match the notion that PLIN5 may be protective, and triggered correlative analysis of changes in PLIN5 with markers of insulin sensitivity.

These correlative analyses revealed no association between changes in PLIN5 and insulin sensitivity in in participants from South Asian origin, who became more insulin resistant after the HFHC-diet, despite a significant increase in skeletal muscle PLIN5 content. This suggests that the previously hypothesized role of PLIN5 in prevention of insulin resistance^15^,^28^,^37^ may operate in Caucasians, whereas in the present study, this was not observed in South Asians. This apparent discrepancy between Caucasian and South Asian subjects in dealing with excess lipids may originate from differences in mitochondrial lipid handling. Thus, we examined markers of fat oxidative capacity and observed that at the whole body level, rates of lipid oxidation under basal as well as under insulin-stimulated conditions were comparable between ethnicities^12^. Markers of muscle mitochondrial biogenesis (PGC1-α) or mitochondrial content also revealed no significant differences between Caucasians and South Asians. Although this is in line with previous reports in literature^3^,^6^, it does not provide an explanation for the observed discrepancy.

It has previously been observed that in the obese state a higher fraction of IMCL is present as peroxidized lipids (4-hydroxynonenal (4-HNE))^38^, a lipid-derived reactive aldehyde with proven insulin-desensitizing properties^39^. Indeed, increased 4-HNE protein adducts have been observed in skeletal muscle of insulin resistant humans^40^. Interestingly, we also observed a tendency for a differential response in 4-HNE levels in South Asians compared to Caucasians, with higher levels of 4-HNE in South Asians upon consumption of a HFHC-diet. In the present study, however, the increase in 4-HNE did not correlate with the changes in insulin-stimulated glucose uptake in any of the groups. Rather, 4-HNE correlated positively with the increase in PLIN5 in South Asians and not in Caucasians. Whether this correlation merely reflects that induction of PLIN5 is paralleled by increases in IMCL (hence rendering the opportunity for more lipids to be subject to peroxidation) or that it is a reflection of a more complex and indirect interaction remains to be elucidated.

In conclusion, the present study shows that despite similar increases in IMCL content, insulin-stimulated glucose uptake in subjects from South Asian origin is compromised more profoundly than in subjects from Caucasian origin. In contrast to our hypothesis, this differential response does not seem to originate from differences in proteins involved in lipid droplet dynamics or fat oxidative capacity. In Caucasians, the LD coat protein PLIN5, which is involved in regulating oxidative lipolysis, seems to be protective against HFHC diet-induced insulin resistance. For reasons not yet known, this potential protective effect of PLIN5 is not observed in South Asians, who do possess higher myocellular levels of insulin desensitizing lipid peroxidation products than Caucasians.

## Methods

### Ethic statement

The Medical Ethic Committee of Leiden University Medical Center approved this study. The study was performed according the principles of the revised Declaration of Helsinki. All volunteers gave written informed consent before participation. The present study represents a detailed analysis of new parameters assessed in samples derived from a previously published paper^12^.

### Subjects and study protocol

Twelve healthy lean Caucasian and twelve healthy lean South Asian males aged between 19 and 25 years were included. The study protocol was described previously^12^. In short, all subjects followed for 5 days a HFHC-diet consisting of their regular diet with the addition of 3 cups of 125 ml cream per day (1275 kcal/day, 94% fat). Before and after the diet a muscle biopsy was taken from the *m. vastus lateralis* with a modified Bergström needle^41^, and a 2-step hyperinsulinemic-euglycemic clamp and indirect calorimetry with a ventilated hood (Oxycon Pro, CareFusion, Höchberg, Germany) were performed. The indirect calorimetry was performed during basal conditions and during both steps of the hyperinsulinemic-euglycemic clamp (for detailed data see ref. 12).

### Triacylglycerol content

Triacylglycerol (TAG) was biochemically extracted according to Schwartz and Wolins^42^ from 5–10 mg muscle tissue. TAG content was corrected for protein concentration, which was determined with the DC kit (Bio-Rad, Veenendaal, The Netherlands).

### Western blotting

Western blots were performed with primary antibodies against ATGL (2138, Cell Signalling Technology, Bioké, Leiden, The Netherlands), CGI-58 (NB110-41576, Novus Biologicals, Littleton, Colorado, USA), PLIN2 (GP40, Progen Biotechnik, Heidelberg, Germany), PLIN3 (M6PRBP1, Acris, Herford, Germany), PLIN5 (GP31, Progen Biotechnik, Heidelberg, Germany), 4-HNE (393207, Calbiochem, VWR International BV, Amsterdam, The Netherlands), PGC-1α (516557, Calbiochem, VWR International BV, Amsterdam, The Netherlands) and an antibody cocktail against distinct structural proteins of the 5 individual mitochondrial electron transport chain complexes (OXPHOS ab110411, Abcam, Cambridge, UK). For ATGL, PLIN2, PLIN5 and OXPHOS detection IRDye700-conjugated or IRDye800-conjugated secondary antibodies were used and visualized with the Odyssey infrared detector (LI-COR Biosciences, Westburg, Leusden, The Netherlands). An HRP-conjugated secondary antibody was used for CGI-58 and detected with ECL. For all antibodies the molecular weight in the gel was checked with known molecular weight and positive and/or negative controls are applied to assess the validity of the band selected for quantification. In the current study we have used HEK cells overexpressing PLIN2 or PLN5 to identify the appropriate band. For PLIN3 we have a synthetic peptide that we run on the same gels to identify band height and for ATGL we used tissue of ATGL ko mice and a patient with a compound ATGL mutation^43^ to identify the right band. For CGI-58 we combined the information of the data sheet of the supplier with data from fibroblasts of a CGI58-deficient patient^14^. For PGC1-α tissue from a PGC1-α transgenic mice was used as a positive control. For valid loading of equal amounts of muscle protein, a three step approach is used; an instant blue gel is loaded allowing us to ensure that we indeed load uncontaminated muscle tissue. Next, this gel is scanned for optical density to calculate how much of the lysate we need to load to a new gel to obtain equal amounts of protein. Thus, the gel loaded for Western blotting has the same amount of protein loaded to each and every lane and is a valid reflection of a comparable amount of muscle protein. To check for efficient electro transfer of the gels to the membrane, we included the data for the housekeeping protein α-SR-actin (A2172, Sigma, St. Louis, USA) or GAPDH (2118, Cell Signaling Technology, Bioké, Leiden, The Netherlands) as reference. For valid inter-blot comparisons, we used the background correction option in the software of the supplier (Image Studio™ Software for the Odyssey CLx - LI-COR Biosciences) and scanned the corresponding band of the protein of interest for all lanes on the blot and compute the mean of the relevant bands. Finally, we compute the ratio of the optical density of individual protein bands over the mean of the proteins bands in that particular blot.

### Statistics

Results are presented as mean ± SEM. Statistical testing was performed with SPSS version 21.0 (SPSS, Chicago, IL, USA). A repeated measures ANOVA with diet as a within factor and group as between factor was performed to test for statistical significant differences between groups and diet effect. When appropriate, a Bonferroni post-hoc test was performed. Pearson correlations coefficients were used to describe the linear association between variables. P < 0.05 was considered statistically significant.

## Additional Information

**How to cite this article**: Gemmink, A. *et al*. Lipid droplet dynamics and insulin sensitivity upon a 5 day high-fat diet in Caucasians and South Asians. *Sci. Rep.* **7**, 42393; doi: 10.1038/srep42393 (2017).

**Publisher's note:** Springer Nature remains neutral with regard to jurisdictional claims in published maps and institutional affiliations.

## Supplementary Material

Supplementary Figures

## Figures and Tables

**Table 1:** Correlations between PLIN5 protein content and markers of insulin sensitivity in Caucasian and South Asian subjects before and after a 5-day HFHC-diet.

		All	Before	South Asians	All	After	South Asians
			Caucasians			Caucasians	
R_d_	*r*	**0.610**	**0.895**	0.172	0.257	**0.668**	0.084
(μmol*L/Kg_LBM_/min/mU)	P	**0.004**	**<0.001**	0.635	0.275	**0.035**	0.818
	n	20	10	10	20	10	10
RQ basal	*r*	0.234	−0.031	0.442	**0.482**	**0.680**	0.391
	P	0.295	0.929	0.174	**0.027**	**0.030**	0.235
	n	22	11	11	21	10	11
RQ insulin stimulated	*r*	0.405	**0.618**	0.328	**0.446**	0.322	0.416
	P	0.076	**0.043**	0.389	**0.049**	0.398	0.203
	n	20	11	9	20	9	11
Metabolic flexibility	*r*	**0.472**	**0.856**	0.013	0.103	−0.305	0.187
	P	**0.035**	**0.001**	0.974	0.666	0.424	0.582
	n	20	11	9	20	9	11
Lipid oxidation	*r*	−0.230	0.137	−0.481	−0.419	**−0.684**	−0.337
	P	0.302	0.687	0.134	0.059	**0.029**	0.311
	n	22	11	11	21	10	11
Insulin stimulated glucose	*r*	0.327	**0.750**	−0.085	−0.109	−0.233	−0.090
oxidation (μmol/kg_LBM_/min)	P	0.159	**0.008**	0.828	0.648	0.547	0.791
	n	20	11	9	20	9	11
Insulin stimulated NOGD	*r*	**0.482**	**0.792**	0.151	0.134	**0.817**	−0.030
(μmol/kg_LBM_/min)	P	**0.032**	**0.004**	0.698	0.572	**0.007**	0.930
	n	20	11	9	20	9	11
