# Supplementary material for: Lipid droplet dynamics and insulin sensitivity upon a 5-day high-fat diet in Caucasians and South Asians
Source: Sci Rep. 2017 Feb 14;7:42393. doi: 10.1038/srep42393 (PMC5307956; doi:10.1038/srep42393)
Supplement: Supplementary Figures [file srep42393-s1.pdf]

Lipid droplet dynamics and insulin sensitivity upon a 5 day high-fat diet in Caucasians and South Asians.

Anne Gemmink[1], Leontine E.H. Bakker[2], Bruno Guigas[3,4], Esther Kornips[1], Gert Schaart[1], A. Edo Meinders[2], Ingrid M. Jazet[2], Matthijs K.C. Hesselink[1*]

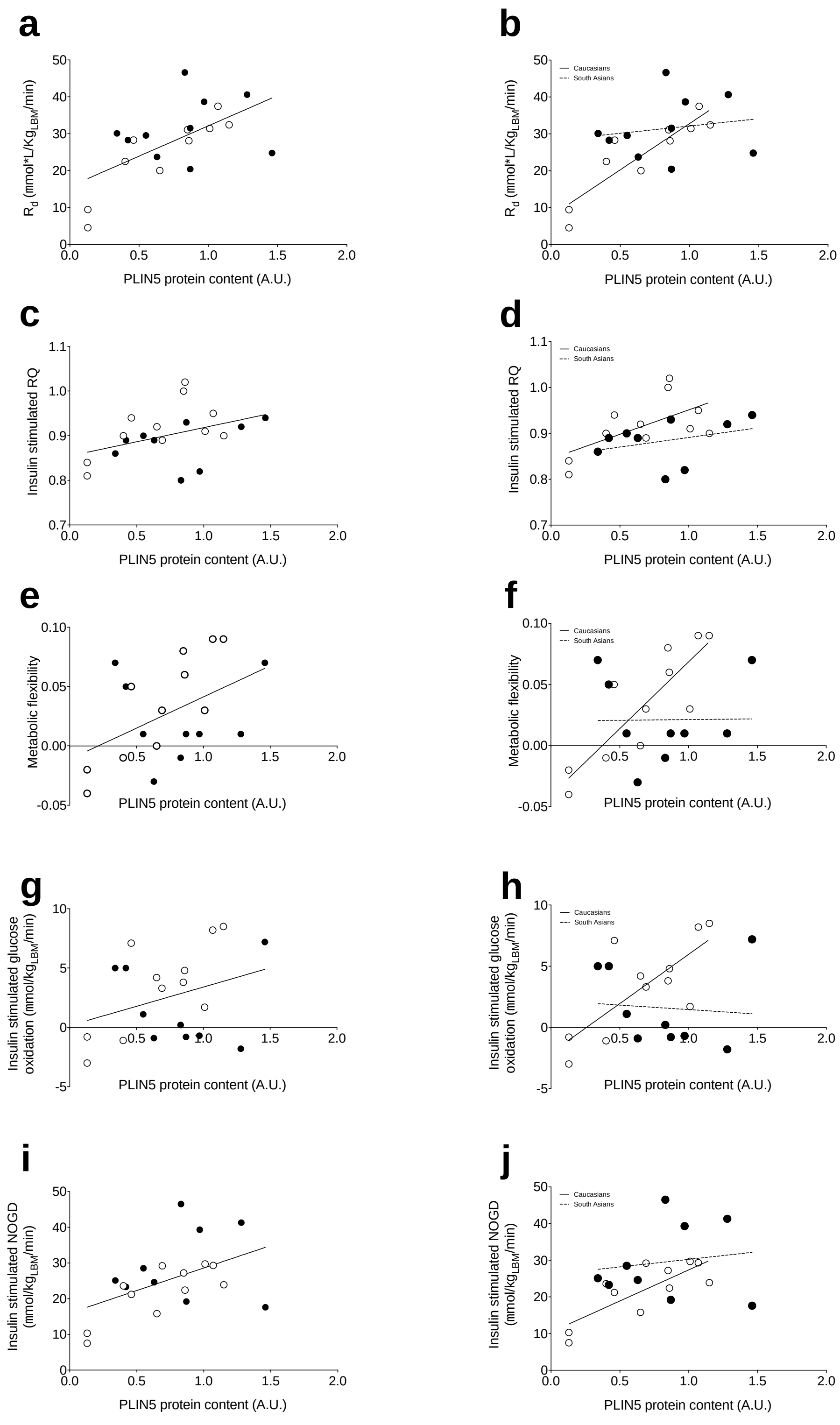

Supplementary Figure S1: significant correlations from Table between PLIN5 and insulin sensitivity markers before the diet. (a, b) PLIN5 vs. glucose disposal. (c, d) PLIN5 vs. insulin stimulated RQ. (e, f) PLIN5 vs. metabolic flexibility. (g, h) PLIN5 vs. insulin stimulated glucose oxidation. (i, j) PLIN5 vs. insulin stimulated NOGD; Caucasians: ○, South Asians: ●.

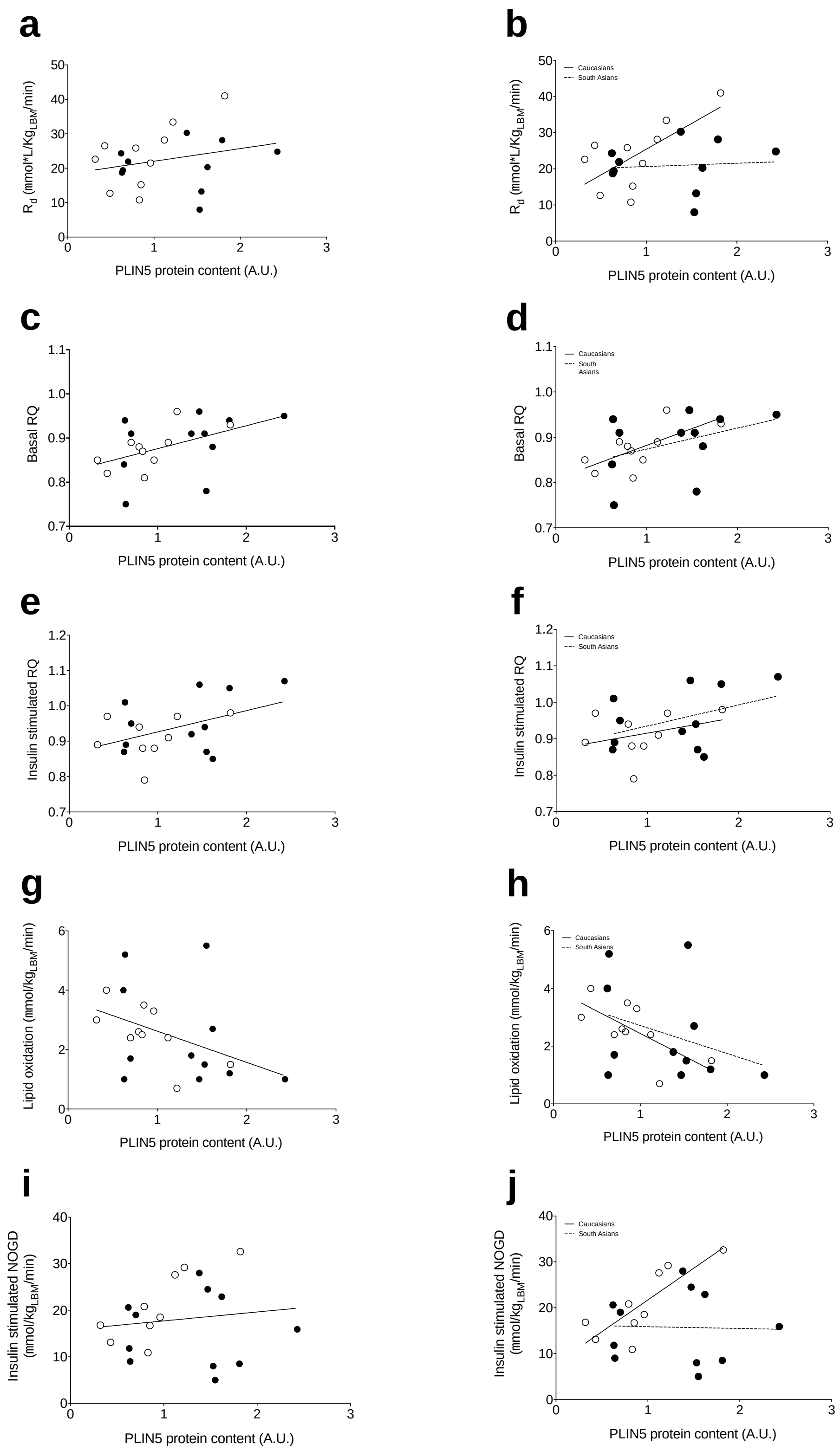

Supplementary Figure S2: significant correlations from Table between PLIN5 and insulin sensitivity markers after the diet. (a, b) PLIN5 vs. glucose disposal. (c, d) PLIN5 vs. basal RQ. (e, f) PLIN5 vs. insulin stimulated RQ. (g, h) PLIN5 vs. lipid oxidation. (i, j) PLIN5 vs. insulin stimulated NOGD; Caucasians: ○, South Asians: ●.
